# Gut microbiota development across the lifespan: Disease links and health‐promoting interventions

**DOI:** 10.1111/joim.20089

**Published:** 2025-04-24

**Authors:** Ida Schoultz, Marcus J. Claesson, Maria Gloria Dominguez‐Bello, Frida Fåk Hållenius, Peter Konturek, Katri Korpela, Martin Frederik Laursen, John Penders, H. Roager, Tommi Vatanen, Lena Öhman, Maria C. Jenmalm

**Affiliations:** ^1^ School of Medical Sciences Faculty of Medicine and Health Örebro University Orebro Sweden; ^2^ School of Microbiology and APC Microbiome Ireland Cork Ireland; ^3^ Department of Biochemistry & Microbiology and of Anthropology Rutgers University–New Brunswick New Brunswick New Jersey USA; ^4^ Department of Food Technology, Engineering and Nutrition Lund University Lund Sweden; ^5^ Department of Medicine, Thuringia Clinic Saalfeld Teaching Hospital of the University Jena Jena Germany; ^6^ Faculty of Medicine University of Helsinki Helsinki Finland; ^7^ National Food Institute Technical University of Denmark Kgs. Lyngby Denmark; ^8^ Department of Medical Microbiology, Infectious Diseases and Infection Prevention, School for Nutrition and Translational Research in Metabolism Maastricht University Medical Center Maastricht the Netherlands; ^9^ Department of Nutrition, Exercise and Sports University of Copenhagen Frederiksberg Denmark; ^10^ Institute of Biotechnology, Helsinki Institute of Life Science (HiLIFE) University of Helsinki Helsinki Finland; ^11^ Department of Microbiology, Faculty of Agriculture and Forestry University of Helsinki Helsinki Finland; ^12^ Research Program for Clinical and Molecular Metabolism, Faculty of Medicine University of Helsinki Helsinki Finland; ^13^ Broad Institute of MIT and Harvard Cambridge Massachusetts USA; ^14^ Liggins Institute University of Auckland Auckland New Zealand; ^15^ Department of Microbiology and Immunology, Institute of Biomedicine, Sahlgrenska Academy University of Gothenburg Gothenburg Sweden; ^16^ Division of Inflammation and Infection, Department of Biomedical and Clinical Sciences Linköping University Linköping Sweden

**Keywords:** adulthood, aging, gut microbiota, infancy, inflammatory diseases, intervention

## Abstract

The gut microbiota plays a pivotal role in human life and undergoes dynamic changes throughout the human lifespan, from infancy to old age. During our life, the gut microbiota influences health and disease across life stages. This review summarizes the discussions and presentations from the symposium “Gut microbiota development from infancy to old age” held in collaboration with the Journal of Internal Medicine. In early infancy, microbial colonization is shaped by factors such as mode of delivery, antibiotic exposure, and milk‐feeding practices, laying the foundation for subsequent increased microbial diversity and maturation. Throughout childhood and adolescence, microbial maturation continues, influencing immune development and metabolic health. In adulthood, the gut microbiota reaches a relatively stable state, influenced by genetics, diet, and lifestyle. Notably, disruptions in gut microbiota composition have been implicated in various inflammatory diseases—including inflammatory bowel disease, Type 1 diabetes, and allergies. Furthermore, emerging evidence suggests a connection between gut dysbiosis and neurodegenerative disorders such as Alzheimer's disease. Understanding the role of the gut microbiota in disease pathogenesis across life stages provides insights into potential therapeutic interventions. Probiotics, prebiotics, and dietary modifications, as well as fecal microbiota transplantation, are being explored as promising strategies to promote a healthy gut microbiota and mitigate disease risks. This review focuses on the gut microbiota's role in infancy, adulthood, and aging, addressing its development, stability, and alterations linked to health and disease across these critical life stages. It outlines future research directions aimed at optimizing the gut microbiota composition to improve health.

## Graphical abstract

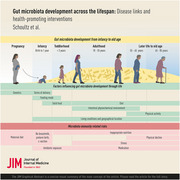

## Introduction

**Fig. 1:**
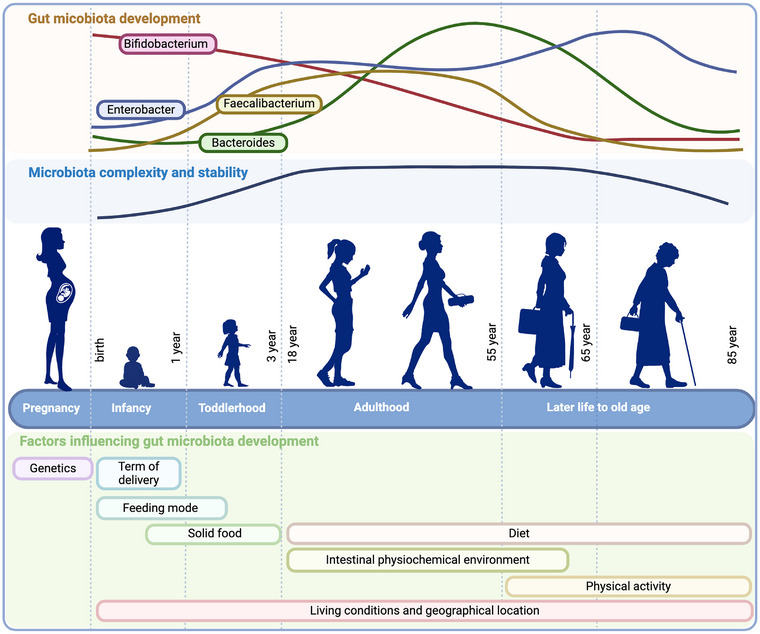
Schematic illustration of the development of the gut microbiota from infancy to old age, including factors influencing the composition of the gut microbiota at different life stages. The top panel shows how four of the dominant bacterial genera, that is, Bifidobacterium, Enterobacter, Faecalibacterium, and Bacteroides, in the human gut microbiota vary in abundance through the different stages of life. The second top panel illustrates the complexity and stability of the gut microbiota through life, whereas the lower panel lists factors known to influence the development of the gut microbiota. Source: Created with biorender.com.

The human gastrointestinal (GI) tract harbors the gut microbiota, a vast and complex microbial community that plays a crucial role in maintaining the host's health throughout the lifespan [1]. From birth to old age, the gut microbiota is shaped by an interplay of genetic, environmental, and lifestyle factors (Fig. 1) [2]. The gut microbiome is not only central to a well‐functioning GI tract but is also known to play an important role in controlling diverse physiological functions, including immune modulation [3], metabolic homeostasis [4, 5], and neurological processes [6, 7]. Thus, the developments of the gut microbiota in infancy, as well as the compositional changes that occur throughout life, have a major impact on health and aging.

The development of the gut microbiota commences at birth, a critical period where the mode of delivery and early feeding practices lay the foundation for microbial colonization [8, 9]. This early microbial assembly sets the stage for subsequent transformations during childhood and adolescence, marked by a steady increase in microbial diversity, impaired by exposure to antimicrobials, and affected by environmental exposures. In adulthood, the gut microbiota establishes a relatively stable state reflecting a delicate equilibrium influenced by environmental exposures, and particularly dietary habits and genetic makeup [1]. Although this phase is characterized by high intra‐individual variation, the resilience of the gut microbiota generally ensures that the intricate interaction with the host is maintained [10, 11]. Advancing age, however, introduces a new dimension to the delicate relationship between the host and the microbiota, marked by a decline in microbial diversity and alterations in composition. This age‐associated dysregulation has been implicated in various health conditions prevalent in the elderly, raising questions about the causative role of the gut microbiota in aging‐related diseases [11].

**Fig. 2:**
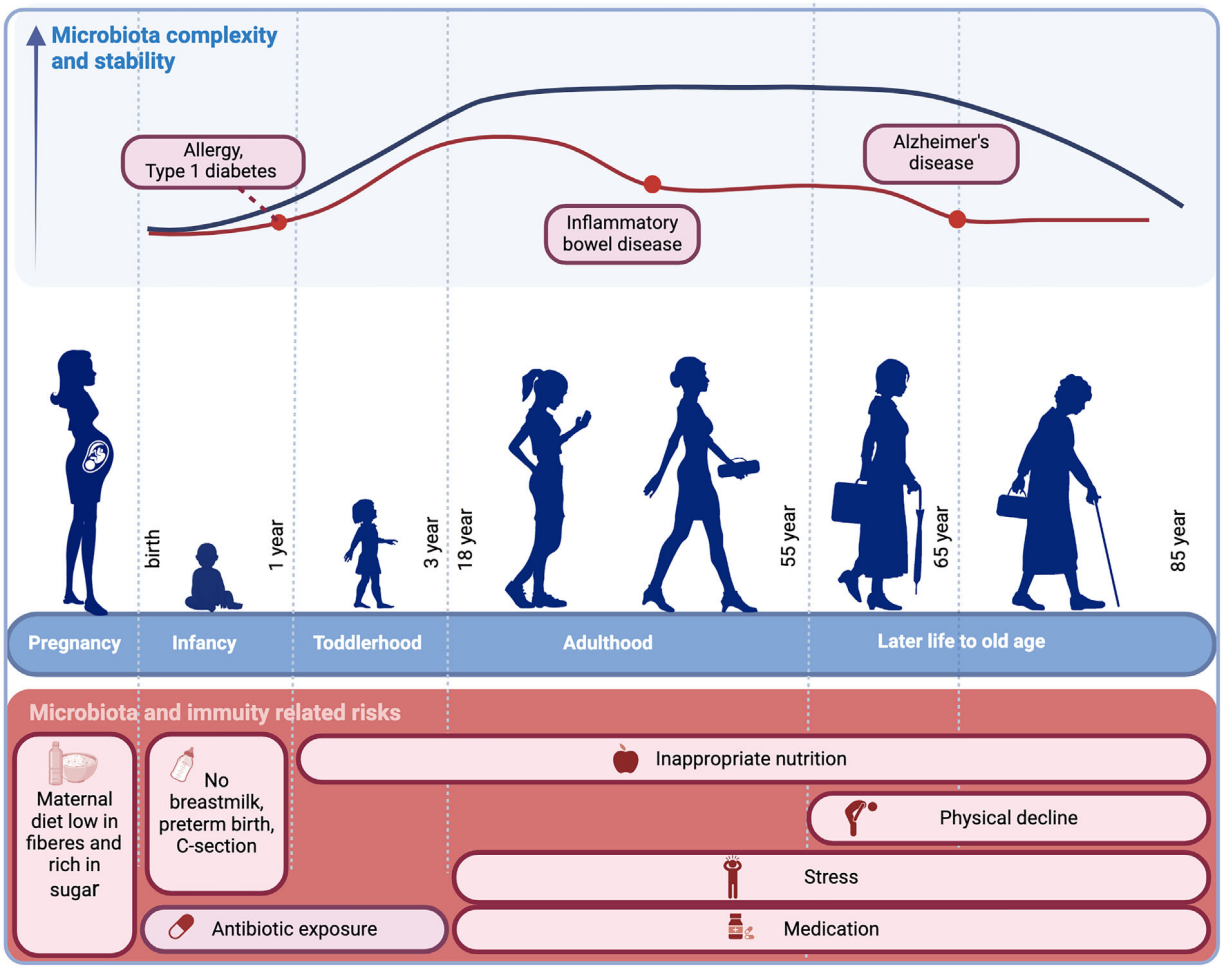
Overview of the variation in complexity and stability of the gut microbiota in relation to risk factors influencing the gut microbiota composition at different life stages. Throughout life, several risk factors are associated with a perturbed gut microbiota composition and potentially the development of several diseases. A reduction in the gut microbiota complexity and stability during infancy has, for example, been associated with the development of allergy and Type 1 diabetes. In adulthood and later life, a perturbation of the gut microbiota has been associated with the development of inflammatory disorders, such as inflammatory bowel disease, as well as the neurodegenerative disease Alzheimer's disease, mainly developing later in life. Source: Created with biorender.com.

To develop effective strategies for restoring or preserving a healthy microbiota, it is imperative to refine our understanding of the processes driving gut microbial assembly as well as understanding the compositional fluctuations of the gut microbiota throughout life [12]. Although substantial progress has been made in deciphering the broad strokes of gut microbiota development, numerous unknowns persist [13, 14, 15, 16]. The intricate details of early life microbial colonization, the functional implications of specific microbial strains, the cause of age‐associated microbiota changes throughout the lifespan, and the precise mechanisms by which the gut microbiota influences neurological, immune, and metabolic health remain areas of investigation [17]. Unraveling these complexities is not only pivotal for understanding fundamental aspects of human biology but also holds promise for developing targeted interventions to promote health and mitigate disease risks across the lifespan (Fig. 2). One of the most extensively studied areas of gut microbiota research is its association with inflammatory diseases. Accumulating evidence has highlighted strong links between disruptions in the gut microbiota and the development of inflammatory conditions [1]. At the symposium that this review summarizes, inflammatory bowel disease (IBD) [18, 19], Type 1 diabetes (T1D) [20, 21], allergic diseases [22], and Alzheimer's disease (AD) [23] were discussed (Table 1).

**Table 1:** Gut microbiota and associations with diseases discussed at the symposium.

Disease group	Key gut microbiota associations
Type 1 diabetes (T1D)	Increased *Bacteroides* spp. [20, 24–27], decreased SCFA‐producing bacteria [20, 21, 28, 29]
Allergy	Low microbial diversity in infancy [30, 31, 32, 33, 34, 35, 36, 37]; and delayed gut microbiota maturation [30, 37–39]
Inflammatory bowel disease (IBD)	Distinct gut microbiota and metabolome profiles [18, 19, 40, 41]
Alzheimer's disease (AD)	Reduced microbial diversity, increased Proteobacteria, and decreased Firmicutes [42, 43, 44, 45, 46]

This review examines how alterations in gut microbiota contribute to immune system dysregulation, chronic inflammation, and disease progression across various stages of life. Specifically, it focuses on the three life stages—infancy, adulthood, and later life—detailing the development, stability, and compositional shifts of the gut microbiota while highlighting its associations with inflammatory and age‐related diseases. Additionally, this review explores potential health‐promoting interventions—including dietary modifications, probiotics, and microbiota‐targeted therapies—to mitigate disease risks and promote gut microbial balance. Finally, we discuss methodological challenges in analyzing the gut microbiota and emphasize the need for continued research to refine microbiome‐based interventions for clinical applications.

## The compositional changes of the gut microbiota throughout life and the impact of diet

### Gut microbiota development during infancy and the influence of diet

The neonatal phase lays the foundation for the assembly, diversification, and maturation of the intestinal microbiota. During this critical time window, the microbiota exerts significant influence on both enteric mucosal tissue and the immune system, yielding enduring effects at both local and systemic levels [47, 48]. A deeper understanding of the mechanisms orchestrating the transformation of the initial microbial inoculum into a highly personalized microbial ecosystem—which stabilizes after the initial years of life—holds profound promise to effectively manage and sustain human health [30, 49, 50]. To develop efficacious strategies for restoring or preserving a healthy microbiota, it is thus imperative to refine our understanding of the processes driving gut microbial assembly.

The assembly process begins at birth with the dispersal of microorganisms mainly from maternal sources (e.g., skin, faces, and vagina) [51]. Continued exposure to live bacteria or spores from the mother and other family members [52, 53], as well as other environmental sources, contributes to the developmental trajectory of the infant gut microbiota. In addition, the colonization process is modified by external (e.g., antibiotics and diet) as well as internal abiotic factors (e.g., oxygen and pH levels in the gut) [8, 54, 55]. In short, the gut microbiota composition is determined by a multitude of factors, including gestational age at birth, mode of delivery, use of antibiotics and other medications, diet, the presence of pets and siblings, as well as host genetics and GI environment [8, 54]. Among these, diet seems to be the strongest currently identified determinant for microbiota composition during infancy [30, 56]. Breastfeeding is pivotal in the establishment of the infant gut microbiota both directly—by dispersal of viable bacteria in breast milk—and indirectly, but more importantly, by stimulating the growth of beneficial bacteria nourished via human milk oligosaccharides (HMOs) and inhibiting the growth of potentially pathogenic bacteria via bioactive components such as milk antimicrobials and immunoglobulins [8]. Most of the initial colonizing species in the gut are gut‐adapted bacteria such as *Bifidobacterium* and *Bacteroides* species of maternal gut origin [9] but may also be found in the vagina and perineum, particularly peri‐ and postnatally [57, 58]. *Bifidobacterium* species, such as *B. longum* subsp. *infantis, B. bifidum*, and *B. breve*, dominate the infant gut microbiota during breastfeeding and as a result keep low pH and low diversity. These taxa encode specific HMO membrane transporters and HMO‐degradation enzymes, enabling them to utilize HMOs [59], resulting in the production of acetate, lactate, formate, and 1,2‐propanediol [60]. The lactate produced can be cross‐fed to lactate utilizers such as the *Veillonella* species, which results in some propionate production [61, 62], in addition to propionate produced by the *Bacteroides* species [63]. Due to the low prevalence and abundance of *Lachnospiraceae* and *Oscillispiraceae* species in the infant gut during breastfeeding, levels of butyrate are limited in this phase. In contrast, formula‐fed (FF) infants have a more diverse microbiota consisting of increased numbers of *Bacteroides*, *Clostridium*, and *Enterobacteriaceae*, including some opportunistic pathogens [30, 64–66]. The gut microbiota of FF infants produce more propionate and butyrate as well as branched‐chain fatty acids, such as isovalerate and isobutyrate, originating from proteolytic metabolism [67], possibly due to the lack of HMOs and higher protein content of formula milk. It should be noted that newer milk formula contains added mixtures of HMOs (HMO‐FF), which have been shown to bring the gut microbiota of HMO‐FF infants closer to that of breastfed infants [68, 69], primarily by increasing the relative abundance of *Bifidobacterium* species. Weaning and the transition to an adult‐like diet increase diversity in the gut [70], *Veillonella* and *Bifidobacterium* decline, *Bacteroides* increase further, and *Oscillospiraceae* (e.g., *Faecalibacterium*, *Ruminococcus*) and *Lachnospiraceae* (e.g., *Blautia* and *Roseburia*) take on a more dominant role within the gut microbiota [30, 71–73]. As many of these bacteria within the two latter families are butyrate producers, a concomitant increase in butyrate levels is observed [74, 75].

Despite the strong influence of delivery mode, siblings, antibiotic and probiotic use, and infant diet [30, 56, 65, 76], the majority of inter‐individual variation in gut microbiota composition and its developmental trajectories remains unexplained. The timing and order of arrival of species into the gut may affect the species composition, a phenomenon known as *priority effects*, which has been revealed for selected taxa in cohorts of both preterm and term infants [77, 78]. For example, the *Bifidobacterium* taxon first arriving at the gut of breastfed infants initially dominates the ecosystem, despite often being a suboptimal HMO‐utilizer (such as *B. longum* ssp. *longum*) and co‐occurring with a later‐arriving *Bifidobacterium* taxon with superior HMO‐utilization capability (such as *B. longum* ssp. *infantis*). With time, however, the breastmilk‐derived HMOs seem to often select for the superior *Bifidobacterium* HMO‐utilizers that will eventually dominate the ecosystem as long as the infant is (exclusively) breastfed [77]. However, such early dynamics may not occur if the infant is not exposed to bifidobacteria through vaginal birth. This suggests that both priority effects and deterministic forces such as diet together shape infant gut microbiota assembly. Future investigations should consider these ecological phenomena together with known deterministic factors but also delve into undiscovered deterministic factors. Examples of the latter are the impact of host factors—such as bile acids [55] and site‐specific defense mechanisms (e.g., secretory IgA, mucus, antimicrobial peptides) [79]—but also the influence of timing, nature, and diversity of complementary food introduction [80] on microbiota maturation processes that have so far been largely unexplored. Integration of metabolomics, metagenomics, and metaproteomics in longitudinal birth cohort studies with frequent sampling to capture dynamics of host–microbial and host–diet interactions [81], combined with mechanistic studies using isolated strains and defined microbial consortia [82], should further our understanding on early life gut microbiota development.

### The gut microbiota in adulthood and the influence of diet–microbiota interactions

During early life, the gut microbiota composition evolves from a simple community—dominated by bifidobacteria—to a more diverse ecosystem, reaching by approximately 3–5 years of age a composition that closely resembles that of adults [50, 83, 84]. As children progress into adolescence, further modifications in gut microbiota composition occur due to hormonal shifts and other pubertal changes [85]. In adulthood, however, the gut microbiota tends to become more stable and differentiated compared to infancy and is mainly dominated by the bacterial phyla Firmicutes, Bacteroidetes, and Actinobacteria (also named Bacillota, Bacteroidota, and Actinomycetota) [1, 12]. However, variations in microbiota composition and function still exist between individuals due to factors such as diet and geographical location [10, 12, 50]. Approximately 23% of the gut microbiome's compositional variation is explained by intra‐individual factors, highlighting its relative stability over time [10]. Despite this stability, inter‐individual variation makes it challenging to define universal microbial markers of health. The stability of the gut microbiota is mainly attributed to the core microbiota, a subset of microbial species that are consistently present across individuals [1, 5]. Although its composition varies based on geography, genetics, and lifestyle, certain bacterial taxa are commonly found in healthy adults [1]. A Swedish longitudinal study showed that taxa such as *Enterobacteriaceae* and *Lactobacillus* exhibit intra‐individual variation independent of geography or age [10]. Additionally, the gut microbiota demonstrates functional redundancy, where different microbial species fulfill similar metabolic roles, ensuring essential functions persist despite environmental changes [5]. The gut microbiota possesses a remarkable metabolic capability, able to transform both host‐derived and dietary elements (such as lipids, carbohydrates, and proteins) into various metabolites, which can either benefit or pose risks to the host [5, 86]. The short‐chain fatty acids (SCFAs)—including butyrate—are known to be important for intestinal homeostasis and are generated through the fermentation of dietary fibers by commensals, such as *Faecalibacterium prausnitzii* [87] and *Roseburia intestinalis* [88].

Diet is the major factor shaping the adult gut microbiome. Long‐term dietary patterns influence microbiota composition, with high‐fiber diets promoting microbial diversity and SCFA production, whereas high‐fat and low‐fiber diets can lead to dysbiosis [89, 90, 91]. In contrast, drastic dietary changes—such as switching between high‐fat/low‐fiber and low‐fat/high‐fiber diets—lead to rapid temporary shifts in the gut microbiota that return to baseline patterns when the dietary intervention ends [92], emphasizing that the gut microbiome composition is rather stable in healthy adults [93] and thus strongly dependent on host‐related factors.

Most dietary intervention studies in adults show modest and individual‐specific effects on the gut microbiome composition [94]. For example, studies [95, 96] in Danish adults—who typically have a high habitual wholegrain intake—showed that a wholegrain‐rich diet had only subtle effects on gut microbiota composition [95], whereas a low‐gluten diet—excluding all foods with gluten‐rich grains (i.e., rye, wheat, and barley)—had more pronounced effects [96]. Complicating the field further, human intervention studies have also found personal microbiome‐dependent responses to similar foods [97], fibers [98, 99, 100], artificial sweeteners [101], and breads [102]. Additionally, research has shown that gut microbiota composition improves predictive models of personal metabolic responses, such as postprandial glucose, insulin, and triacylglycerol levels [103, 104, 105].

Beyond diet, physiological factors, such as intestinal pH, transit time, and oxygen levels, determine the growth condition for the resident microbes [106, 107]. Recent studies suggest that intestinal transit time may explain more variation in the microbiota composition than diet alone [108, 109]. Longer transit time has been associated with increased proteolytic fermentation, whereas shorter transit time favors saccharolytic fermentation [110, 111, 112]. Enterotypes—which represent preferred gut microbial community structures [113]—have been linked to transit time [114] and different responses to fiber‐rich dietary interventions [115].

Recent research also emphasizes the concept of microbiota resilience, or the ability of the gut microbiota to return to a stable state after perturbations. Although dietary interventions can induce shifts in microbial composition, the gut microbiota often reverts to its original state unless dietary changes are sustained over time. This suggests that long‐term dietary habits, rather than short‐term interventions, are more likely to induce lasting microbiome changes that promote health [10].

Future research should move beyond merely profiling microbiota to studying microbial activity within the intestinal environment. This can be done by combining SmartPills, which measure whole‐gut and segmental intestinal pH and transit time [116]; novel sampling devices that allow sampling throughout the GI tract [117]; and metabolomics [118, 119]. Altogether, this will enable the study of personal diet‐microbiota interactions in the small intestine, proximal, and distal colon, respectively, with emphasis on the intestinal environment and microbial metabolism.

### The aging gut microbiota—a decline in microbial and dietary diversity

The gut microbiota undergoes significant changes as we age, becoming less diverse and more variable between individuals compared to adulthood [11, 120–122]. Several factors, such as the individual aging process, hormonal changes, diet, and geographical location, influence the microbial composition in elderly individuals. In women, menopause has a particularly profound impact on the gut microbiota composition [123], leading to a reduced microbial diversity that may be associated with an increased risk of conditions such as obesity and cardiovascular diseases (CVD) [124].

Additionally, our intestines undergo several physiological changes that can have adverse effects on the gut microbiome. A decline in muscle function affects the alimentary tract and results in reduced motility, which could lead to a slower transit time and constipation [11, 125]. The physiological decline associated with aging further results in a reduced production of digestive enzymes, an impaired swallowing reflex, and altered sensation [125]. These changes often lead to altered nutrition dynamics and diets that, in combination with the senescence‐induced “inflammaging,” impact the gut microbiome in noticeable ways [126, 127]. Inflammaging occurs as a normal part of the aging process and results in a chronic, low‐grade inflammation. Several factors contribute to inflammaging, including age‐associated microbial shifts in the gut microbiota composition. Thus, as we age, there is a gradual increase in inflammatory markers and sustained activation of the immune system [128]. The composition of the gut microbiota, therefore, plays a crucial role in healthy aging as it transduces environmental signals, including host immune function as well as metabolic and neurological function, and modifies the risk of age‐related diseases [11]. In addition, the microbiome has a reciprocal relationship with age, changing as the host ages, and is further altered in age‐related diseases, while also modifying age‐related impairment of the host. Investigating the composition of the gut microbiota in healthy aging in a confounder‐free way is therefore challenging. Centenarians are one of the models that have been used for studying healthy aging due to their ability to reach advanced ages while maintaining relatively good health and functional independence [129]. Such findings have shown that centenarians exhibit a more diverse gut microbiota compared to younger individuals and non‐centenarian elderly [130, 131]. Their gut microbiota also often exhibit characteristics associated with better gut health, such as higher abundance of beneficial bacteria (e.g., *Bifidobacteria* and *Lactobacilli*) [132]. However, it is important to note that not all centenarians are free from age‐related diseases or disabilities. Senior orienteering athletes have emerged as a potential model of healthy aging, experiencing fewer GI symptoms and a higher degree of well‐being compared to the general elderly population [133, 134, 135]. Studies in senior orienteering athletes have shown that exercise is associated with alterations in the gut microbial composition, including increases in beneficial bacteria such as *Akkermanisa muciniphila* and *F. prausnitzii* [133]. However, the extent to which these changes in the gut microbiota promote health is not fully understood.

Two seminal studies from the Irish ElderMet project characterized the fecal microbiome in the largest to date longitudinal study, incorporating health indicators such as residence, dietary habits, cognition, frailty, and inflammatory markers [126, 127]. There does not seem to be a particular age threshold at which the microbiota suddenly changes; rather, this happens gradually with time. The microbial composition and diversity proved to be relatively stable over time, but it was also characterized by unusual phylum proportions and extreme variability in some individuals [126]. Moreover, the older persons could be separated by residence location based on these data, particularly into either community or long‐term care [127]. Living in the community was associated with significantly higher microbial and dietary diversity, where the diet appeared to be driving, or at least contributing to, the changing gut microbiota when moving into care homes. As perhaps expected, long‐stay care residents were also frailer and had higher levels of inflammatory markers and fewer SCFA‐producing bacteria in their stool. These microbial and population structures provide a framework for deeper investigations of microbiota‐health associations and causality [121], with eventually even more granular grouping definitions [136]. Based on this framework, adjusting for age could also allow for better identification of microbiome associations with a number of non‐communicable diseases [137].

## The role of the gut microbiota in the development of inflammatory diseases throughout life

Not only does the gut microbiota play a crucial role in human health across the life span, but it has also been implicated in the development of a diverse range of inflammatory diseases, including diabetes, allergies, IBD, and AD [1]. Here, we discuss the dynamic relationship between the gut microbiota and diseases that develop in different stages of life, shedding light on the mechanisms by which alterations in the gut microbiota composition can contribute to the development and progression of inflammatory conditions.

### The influence of the infant gut microbiota in the development of Type 1 diabetes

The gut microbiota has emerged as a key player in the development and progression of autoimmune diseases. Among these, T1D (e.g., autoimmune diabetes where the islet cells of the pancreas are destroyed by the immune system) has received prominent attention, and gut microbiome changes in connection to T1D have been reported in many human cohort studies in predominantly the Western population [20, 21, 24–26, 138–142]. Controlled experiments in the non‐obese diabetic (NOD) mouse model have further demonstrated that the gut microbiome harbors both protective and harmful features that may influence the T1D disease process [28, 143–147]. Common findings from these studies are, for example, that individuals who later developed T1D had increased abundance of *Bacteroides* spp. [20, 24–27] and a decrease of certain bacteria producing SCFAs [20, 21, 28, 29]. The DIABIMMUNE study found that *Bacteroides* spp. harbored immunologically silent forms of lipopolysaccharide (LPS) that—unlike immunostimulant *E. coli* LPS—did not protect NOD mice from T1D [147], suggesting that early exposures to LPS have important roles in immune system development and may also modify an individual's risk of developing T1D. SCFAs (acetate, propionate, and butyrate) have a multitude of benefits to gut health that include protection from future diabetes in the NOD mouse [28] and improved glycemic control in humans with T1D [29]. In the NOD mouse, acetate reduced the number of autoreactive T cells in lymphoid tissues, whereas butyrate increased the function and quantity of regulatory T cells (that help control the immune response) [28]. In addition to these specific mechanisms, both epidemiological and metagenomic evidence from the TEDDY study suggest that neonatal probiotics may also reduce the risk of T1D [21, 148]. Although there is still much to learn about the differences among various probiotic strains, *B. longum* subsp. *infantis* (*B. infantis*) stands out. This subspecies has a unique ability to establish itself in the infant gut during breastfeeding, even if other bacteria colonized first [77, 149, 150]. Although *B. infantis* is ubiquitous in many non‐Western populations [151, 152, 153], its prevalence is currently low in many Western populations [21, 154, 155]—even though it has likely been historically more prevalent in Western populations as well [156]. It has therefore been hypothesized that early supplementation with *B. infantis* could provide protection against T1D [155, 157, 158]. An ongoing multicenter randomized placebo‐controlled GPPAD‐SINT1A trial was set up to test this hypothesis [158].

The gut microbiome also plays a role in the progression of T1D after the diagnosis [26, 29, 159–161]. Data from humans with T1D link the gut microbiome composition with host glycemic control [29, 161, 162]. A preliminary study using fecal microbiome transplant (FMT) in newly diagnosed T1D patients showed that this treatment could stop the decline of endogenous insulin production [163]. These emerging data hint at the potential to use microbiome therapies to interfere with T1D progression, potentially slowing it down. Further research and experiments are crucial to pinpoint specific microbial strains, substances, and other microbiome features that relate to host responses and markers associated with T1D.

### The influence of the infant gut microbiota on allergy development

The increasing prevalence of allergic and other immune‐mediated diseases in affluent countries—where environmental exposures and lifestyles have rapidly diverged from those with which humans evolved—may be caused by reduced diversity of microbial stimulation [22]. The gut microbiota, derived from the mother at birth and from family members postnatally, may provide crucial signals shaping the development of a balanced postnatal innate and adaptive immune system, including appropriate maturation of regulatory T‐cell responses and establishment of adequate mucosal barrier function [47, 164]. Early compositional and functional differences in infancy have been observed to precede the onset of immune‐mediated diseases, including allergic diseases, suggesting that adverse influences on the establishment of the gut microbiota may have long‐lasting consequences [22, 164].

Although early gut microbiota differences between infants who later develop or do not develop allergic disease have been reported in several longitudinal studies [22, 30–39, 47, 164–171], there is considerable inconsistency between the results from different studies, as well as large differences in the study designs and techniques employed. Although no specific microbes with consistently harmful or allergy‐protective roles have yet been identified, some studies have found associations between low rates of colonization with immunomodulatory *Bacteroides* taxa during infancy and later allergy development [32, 35, 169–173]. Early establishment of a diverse gut microbiota—providing repeated exposure to new bacterial antigens and a consistent immunomodulatory impact—may be more important than the distribution of specific microbial species in shaping normal mucosal and systemic immune maturation, and several studies have demonstrated a low gut microbiota diversity during infancy in children later developing allergy [30, 31, 32, 33, 34, 35, 36, 37]. In‐line with impaired mucosal immune maturation increasing allergy risk, aberrant IgA responses to the gut microbiota during infancy preceded allergy development [174]. Furthermore, delayed gut microbiota maturation during infancy has been associated with later allergy development [30, 37–39].

The potential benefits of probiotic supplementation—defined as live microorganisms that confer health benefits when consumed in adequate amounts—in targeting the gut microbiota for allergy prevention have been evaluated in several randomized placebo‐controlled clinical trials [175]. A benefit of probiotics for primary prevention of atopic eczema has been reported in meta‐analyses [176, 177], and a combined pre‐ and postnatal supplementation seems to be crucial for the preventive effect [164, 175–177]. However, due to the large heterogeneity in the design of the clinical trials, the evidence cannot be translated into specific practice guidelines on the most effective probiotic strains, dosages, or optimal duration of treatment [175]. Moreover, probiotic interventions have so far failed to prevent asthma [164, 175]. Further studies on the appropriate timing of interventions and the complex interactions between the infant immune system and the gut microbiota are required to identify preventive strategies to combat the asthma and allergy epidemic.

### The role of the gut microbiota in the development of gastrointestinal diseases in adulthood

Diseases of the GI tract have many challenges. First, diseases such as IBD are often cumbersome to diagnose, and patients must undergo troublesome examinations, that is, colonoscopies. Second, after being diagnosed, the disease is difficult to monitor as the intestinal tissue does not allow for non‐invasive visual examinations, and there is a lack of disease‐specific biomarkers. Third, the exact mechanisms for the first onset of disease or triggers of flares of established disease are still to be understood. The gut microbiota and the metabolites associated with IBD show the potential to, at least partly, solve these challenges.

Numerous studies have demonstrated that patients with IBD have a distinct gut microbiota, often demonstrated by the microbiota composition in fecal samples, but reported associations vary widely across studies [18, 19, 40, 41]. There are reports of the gut microbiota to be highly stable in IBD patients before, during, and after exacerbation [30, 178], whereas other longitudinal analyses reveal reduced temporal microbiota stability in IBD, particularly in patients with changes in disease activity [18].

Not only the composition of gut microbiota but also the products of their metabolism—that is, metabolites—are key factors for GI diseases. The intestinal metabolite profile reflects the interaction between the microbiota and host metabolism, and an altered fecal metabolome has been linked to numerous disorders, including IBD, irritable bowel syndrome (IBS), and colon cancer [179, 180, 181]. It has also been demonstrated that the total metabolome differs between patients with colon cancer, ulcerative colitis, IBS, and healthy subjects [182]. Interestingly, the fecal metabolome has been demonstrated to be stable over time within IBS patients as well as healthy subjects [183]. This supports the concept of a stable fecal metabolome despite potential fluctuations in stool consistency, as well as its potential as a non‐invasive diagnostic tool.

Recently, in vitro studies have shown that the intestinal luminal content—including metabolites—regulates epithelial layer responses to harmful stimuli and possibly promotes disease. When compared to healthy subjects, fecal luminal content from IBS patients induced a distinct colonic epithelial gene expression to intestinal healthy‐derived colonic organoids, potentially reflecting the disease pathophysiology of the fecal donor [184]. The developed experimental model may indeed facilitate the exploration of disease‐related intestinal microenvironmental and barrier interactions. This is further supported by a novel report showing that the stimulation of Caco‐2 cells and human colonic organoids with fecal supernatants derived from patients with colon cancer, ulcerative colitis, and IBS altered the gene expression profiles, potentially reflecting the luminal microenvironment of the fecal sample donor [182]. This experimental approach facilitates the exploration of crosstalk at the gut barrier and the impact of the gut microenvironment on the development of intestinal diseases. Overall, the intricate interplay among microbiota composition, their metabolites, and other luminal factors with the host is essential not only for maintaining immune homeostasis but also for influencing the host's susceptibility to intestinal diseases.

### The role of the gut microbiota in the development of Alzheimer's disease later in life

Emerging evidence suggests that alterations in gut microbiota composition may play a significant role in the pathogenesis of neurodegenerative disorders commonly developing later in life such as AD and Parkinson's disease [23]. Although the gut microbiota undergoes several changes during the normal aging process, the gut microbiota composition in patients with neurodegenerative diseases displays a reduced microbial diversity and a shift in the abundance of specific bacterial taxa [185].

AD is the most common neurodegenerative disorder, resulting in a gradual cognitive decline, memory loss, and changes in behavior and functioning. In patients with AD, studies have shown that the gut microbiota composition appears to differ from that of healthy elderly individuals [42, 43, 44, 45, 46]. However, the diverse nature of sequencing techniques, DNA extraction protocols, and patient stratification makes comparisons between studies difficult. The main robust differences are an increased abundance of Proteobacteria (also named Pseudomonadota) and decreased abundance of Firmicutes in patients with AD [42, 43, 44, 45, 46]. Furthermore, the abundance of five different gut bacterial taxa (*Erysipelatoclostridiaceae*, *Erysipelotrichales*, *Patescibacteria*, *Saccharimonadales*, and *Saccharimonadi*) gradually increases in patients with mild cognitive decline to patients with AD, suggesting that these five taxa may affect progression of symptoms [45]. Another study also found increases in gut *Erysipelotrichaceae* in AD [46]. This taxon has been associated with CVD and high‐fat feeding in mice, and it may affect AD through its relation to CVD, a known risk factor for AD.

As observational studies indicate associations between the gut microbiota and AD, the use of antibiotics has been evaluated. However, no clear evidence for the prevention or treatment of AD with antibiotics has been established in humans. In animal models, antibiotics have been able to affect both pro‐ and anti‐inflammatory cytokines and the progression of amyloid‐beta plaques in brain tissue [186]. It is plausible that antibiotics can indeed affect AD, but only in the more stringent setting of a mouse model, with a less diverse gut microbiota and a controlled diet and environment. In humans, antibiotics are difficult to administer over the long course of disease development, with many side effects, and the impact of AD remains elusive. For efficient gut microbiota modulation in humans, more targeted bacterial therapies are likely needed. Here, specific dietary factors may be developed to maintain a healthy gut microbiota throughout life in at‐risk individuals. A direct causality for microbial involvement in AD has been shown in mice using germ‐free mice and fecal microbiota transplants [186]. The altered microbiota found in 8‐month‐old APPS21 mice with AD pathology led to a quicker progression of AD pathology in the brain upon transfer to germ‐free recipient mice than transfer of microbiota from healthy, wild‐type mice [186]. In conclusion, the current findings point toward an important role for gut microbiota in development of AD. Still, the long time during which AD progresses—several decades—makes preventative measures targeted at gut microbiota modulation challenging. Risk factors for AD, such as age, CVD, and genetic factors, may in part be mediated by gut microbiota shifts, and specific dietary factors may be identified in the future that could prevent such changes in the gut microbiota, also over a long timespan.

## The potential of health‐promoting gut microbiota interventions in different stages of life

The pivotal role of the gut microbiota in maintaining human health has led to an emerging interest in targeted interventions aimed at modulating the gut microbiota to promote health and prevent disease across the lifespan. Understanding the dynamic interplay between the gut microbiota and host physiology at different life stages offers promising opportunities for developing personalized and effective strategies for health promotion. Here, we explore the potential of gut microbiota interventions in different stages of life encompassing infancy, childhood, adulthood, and old age (Table 2).

**Table 2:** Current microbiota‐targeted therapies across different life stages.

Life stage	Probiotics	Prebiotics	Dietary interventions	Transfer of maternal microbes	Fecal microbiota transplantation
Infant	Used for eczema prevention and immune modulation; no clear evidence for asthma prevention [175, 176, 177]	Enhancing gut microbiota diversity to reduce risk of allergies and autoimmune diseases; promising but inconclusive [22, 164, 175]	Breastfeeding and dietary supplementation with *Bifidobacterium infantis* linked to immune benefits [54, 56, 77, 187, 188]	Restoration of microbiota in C‐section infants; limited evidence but gaining interest [189, 190, 191]	N/A
Adulthood and later life	Support microbial diversity; inconclusive effect on immune function [192, 193, 194, 195]	Aging‐associated microbiota changes targeted by prebiotics; promising but inconclusive [196, 197, 198, 199, 200]	Dietary strategies, including traditional Mediterranean diet, were investigated for reducing gut microbiota dysbiosis in aging [201, 202]	N/A	FMT established for *Clostridioides difficile* infections; under study for GI and metabolic diseases [203, 204, 205, 206, 207, 208, 209], Alzheimer's and Parkinson's disease [186]

### Providing maternal microbes to caesarean section‐delivered neonates

The passage of microbes from mothers to their offspring is a widespread and evolutionarily conserved phenomenon [210]. The proximity of perineal reproductive and fecal excretion canals is not a random design from nature. Reptiles, birds, and monotreme mammals with a cloaca where both functions are performed, as well as fish and mammals with adjacent location of birth canal and rectum, suggest facilitation of exposure to both vaginal and fecal bacteria during natural birth [210]. This vertical transfer may play a critical role in evolutionary development by providing a genetically tailored microbiota and optimal mutualism [164]. Moreover, microbial transmission is accompanied by maternal immune‐modifying factors, such as antibodies transferred via the placenta and breast milk [164]. Delivery by caesarean section (CS) disrupts these opportunities for the pioneer microbial colonizers to be transferred from the mother to her baby. This disruption occurs in a developmental window during which immune system‐microbial interactions are critical for normal immune maturation [164].

The disruptions in infant gut microbial ecology caused by CS delivery include a strong depletion of the immunomodulatory genus *Bacteroides* [9, 30, 54, 56, 57, 65, 72, 166, 187, 211–214]. Although this depletion typically resolves after the first year of life, a decreased diversity within the Bacteroidetes (also named Bacteroidota) phylum in CS‐delivered infants was still observed at 2 years of age [211]. Interestingly, maternally acquired strains persisted in the infant's gut to a much higher extent than strains acquired from other sources, in‐line with the theory that maternal strains are optimally adapted for survival in the offspring [51]. Reduced *Bifidobacterium* colonization is also observed after CS delivery [9, 54, 166, 187, 189, 212–214]. However, sometimes *Bifidobacterium* species may be maternally transmitted during breastfeeding [187, 188, 215, 216], although breastfeeding does not restore the natural levels of bifidobacteria in CS‐born infants, despite bifidobacterial growth promoted by the HMOs present in breast milk [54, 56, 77, 187, 188]. Another issue with CS delivery is the increased risk for transmission of opportunistic pathogens—such as *Enterococcus*, *Enterobacter*, and *Klebsiella* species—not from the mother, but from environmental sources, including the hospital [214]. Increased colonization with the opportunistic pathogen *Clostridioides difficile*—which expands when gut microbiota niches are vacant [217]—has also been reported among infants born by CS in several studies [54, 165, 166, 212].

There is a worrying global increase in the frequency of CS deliveries, with rates quadrupling since 1970, and CS deliveries are performed in proportions beyond necessity [218]. There are multiple indications for CS, including cases involving maternal health, fetal distress, or malpresentation, with an estimated need of at least 10% of births [219]. However, rates surpass 50% in many countries, where CSs are performed by choice, without clinical indication [219, 220], likely for doctors’ and hospitals’ convenience, and due to the mother's traumatic labor experiences during vaginal birth. According to studies, 20%–98% of mothers globally face violence or inhumane treatment during childbirth [221, 222, 223]. In order to promote vaginal birth, it is important to change attitudes and practices that render giving birth a traumatic experience.

Birth by CS has been associated with the development of immune‐mediated diseases, including allergy, asthma, Type 1 diabetes, and celiac disease [224, 225, 226, 227, 228]. The disturbed microbial colonization patterns in CS‐delivered infants may thus impair healthy immunological programming. Therefore, it is important to identify methods to restore the natural primary microbiome in these infants and carefully evaluate potential health benefits versus risks. In a 1‐month follow‐up study, in which vaginal microbes were transferred to the neonate (“vaginal seeding”), the gut microbiota of the infants was affected but not influenced by the treatment to the same extent as the skin and oral microbiota [189]. Importantly, vaginal derived facultatively anaerobic lactobacilli may be absent from the colon but thrive in the ileum [229, 230], where important immune interactions are likely to occur [230, 231]. However, ileum samples are invasive and not possible to obtain in healthy infants for ethical reasons. Regarding the strong depletion of immunomodulatory *Bacteroides* taxa observed in CS‐delivered infants, it is important to note that maternal stool is the most likely source of these *Bacteroides* species. In a proof‐of‐concept pilot study, maternal FMT corrected the persistent lack of *Bacteroides* taxa in seven CS‐born infants [190]. An alternative strategy may be to provide both maternal vaginal and fecal microbes to CS‐delivered neonates to more closely approximate the natural inoculum received by neonates during vaginal delivery. These strategies need to be evaluated in institutional review board‐approved randomized clinical trials that are carefully monitored and adequately powered, with appropriate primary clinical outcomes, careful screening for maternal gut and vaginal pathogens, and detailed characterization of the gut, oral, and skin microbiome development during infancy. In the RoMans trial randomized placebo‐controlled trial, 330 families are recruited, and the outcome is IgE‐mediated disease during the first 2 years of life. The results of this and other randomized clinical trials will determine the degree of safety and disease protection by providing maternal microbes to CS‐delivered neonates [191]. In parallel, more stewardship and education to humanize the experience of natural birth and reduce unnecessary CS deliveries is of utmost importance.

### The potential of fecal microbiota transplantation in gastrointestinal diseases

FMT is defined as an administration of fecal material containing gut microbiota from a healthy person (donor) to a patient (recipient). This method allows replacing an unfavorable gut microbiota with a presumably favorable one from a healthy donor. The main rationale behind this idea is to restore a healthy gut ecosystem that provides critical ecosystem services to the host by regulating the immune system, metabolism, and neurology [232]. The preparation and use of FMT for modulation of gut microbiota include many steps such as patient selection and enrollment, recruitment of the proper donor, stool collection, testing and processing of fecal material, FMT procedure, and follow‐up evaluation [233]. FMT can be applied by different routes to the GI tract using upper or lower endoscopy, intestinal tubes, or capsules [234].

FMT is primarily employed in the treatment of recurrent *C. difficile* infection, a disease with a high burden of morbidity and mortality. Importantly, FMT not only prevents the recurrence of *C. difficile* but also significantly reduces the CDI‐associated mortality in patients with fulminant forms of this infection [203]. However, its efficacy in patients with IBD and concurrent *C. difficile* infection may be limited, and multiple FMTs may be required for resolution [204].

In patients with IBD, particularly ulcerative colitis, repetitive FMT shows promise as a therapeutic intervention. Benefits may include the correction of alterations in the gut microbiota, improvement in mucosal barrier function, anti‐inflammatory effects, and partial reversion of intestinal dysfunction [205]. Recently, it has been shown that the response to FMT in patients with ulcerative colitis depends on specific characteristics of the donor microbiota, including high diversity, the presence of specific taxa such as *Roseburia inulivorans* or *Anaerobutyricum hallii* (previously named *Eubacterium hallii*), and the presence of specific metabolites such as SCFA [206]. The efficacy and safety of FMT as a treatment option have been studied in randomized controlled trials. In a recent meta‐analysis, including 13 randomized controlled trials (580 patients, 293 treated with FMT vs. 287 control subjects), it was demonstrated that FMT was associated with higher rates of clinical and endoscopic remission compared to non‐FMT control groups, with no significant differences in adverse reactions [207]. FMT has also been investigated as a potential treatment for IBS, a common clinical condition characterized by symptoms of recurrent abdominal pain associated with changes in bowel motility. Although the exact mechanism behind IBS remains unknown, it is clear that this functional GI condition represents a malfunction in the microbiota–gut–brain axis linked to alterations in the gut microbiota [208]. However, results from randomized controlled studies are inconsistent, possibly due to variations in FMT procedures and donor selection [209]. Further research is needed to fully elucidate its efficacy and mechanisms of action in this context.

In summary, FMT represents a promising frontier in medicine, offering novel insights into the complex interplay between the gut microbiota and human health. Although considerable progress has been made in elucidating its therapeutic potential, numerous questions and challenges remain. Future research efforts are needed to optimize FMT protocols, expand its indications, address safety concerns, and further elucidate its mechanisms of action across a diverse range of diseases and conditions. Through continued investigation and innovation, FMT holds the promise of revolutionizing the management of GI and systemic disorders, ultimately improving patient outcomes and quality of life.

### Gut microbiota interventions targeting the elderly population

Interventions targeting the gut microbiota, including probiotics, prebiotics (i.e., non‐digestible fibers that selectively stimulate the growth and activity of beneficial gut bacteria), and dietary modifications, have emerged as promising strategies to promote health and well‐being in elderly individuals [196, 197]. With aging, the gut microbiota undergoes significant changes in composition, diversity, and functionality [121, 126], which have been linked to various health conditions, including cognitive decline and inflammatory diseases [1, 5, 122]. The instability of the gut microbiota in the elderly further represents a window of opportunity for targeted interventions aimed at restoring microbial balance and enhancing overall well‐being.

Probiotics, particularly strains of the genera *Lactobacillus* and *Bifidobacterium*, have been widely studied for their ability to support microbial balance and reduce inflammation [1, 192–195].

However, results from clinical trials enrolling elderly individuals are inconsistent [192]. Although most studies performed in recent years show an effect on the microbial composition after probiotic supplementation in the elderly, only modest effects have been reported on immune function. One of the challenges in probiotic research among the elderly is the heterogeneity of the population, making it difficult to identify a uniform group for microbiota studies. Additionally, the timing of microbiota decline varies among individuals, complicating research efforts [11]. Beyond general gut health, probiotics have also shown potential in managing menopausal changes, as declining estrogen levels lead to shifts in microbiota composition, reducing microbial diversity and increasing the risk of metabolic and cardiovascular disorders and osteoporosis. Specific probiotic strains of *Lactobacillus* and *Bifidobacterium* may help to restore microbial balance, although more research is needed to establish clear therapeutic guidelines for their use in this context [235].

Prebiotics have also been investigated through clinical intervention studies as a potential modulator of the gut microbiota in elderly individuals [193, 197, 198–200]. Upon reaching the colon, prebiotics are fermented, leading to the production of SCFAs, such as butyrate, which play a crucial role in maintaining intestinal barrier integrity, modulating immune function, and reducing inflammation [197, 200, 236]. Several clinical studies have examined prebiotic supplementation in older adults with mixed results. Although many studies report an increase in beneficial gut bacteria, the impact on broader health outcomes—such as frailty and immune function—remains unclear [196, 198, 237]. Notably, a recent randomized controlled trial using a twin model demonstrated that prebiotic supplementation increased gut microbiota diversity and improved cognitive function in elderly participants [238].

Targeted dietary interventions have also been explored as a potential tool to support gut microbiota and promote healthy aging. For example, the consumption of polyphenol‐rich foods, such as blueberries, has been associated with a moderate increase in microbiota diversity and beneficial bacteria, particularly in older individuals [201]. A more comprehensive approach has been examined through studies on the traditional Mediterranean diet, which is rich in fruits, vegetables, olive oil, and nuts. A large study involving over 600 older adults found that adherence to this diet reduced bacteria linked to inflammation, frailty, and cognitive decline [202]. Although strict adherence to a Mediterranean diet may be challenging for some elderly individuals, modifying dietary patterns to include similar nutrient‐rich components can help counteract age‐related changes in microbiota. Additionally, a study in humanized mice comparing high‐fat/low‐fiber diets (representative of long‐term care settings) with low‐fat/high‐fiber diets (representative of community‐dwelling elderly) found that long‐stay diets in the community mice led to altered immune and microbiota markers associated with higher frailty [239].

An independent systematic review of 27 studies focusing on older populations concluded that longevity is associated with increased microbiome stability and resilience [122]. The authors suggested that healthy aging depends on maintaining anti‐inflammatory activity, facilitated by a high level of SCFA‐producing gut bacteria, despite the age‐related predisposition to an increase of pro‐inflammatory cytokines. To fully harness the benefits of microbiome‐targeted therapies, large‐scale, well‐characterized, and longitudinal multi‐omics studies are needed [11]. Future interventions should aim to personalize strategies based on individual microbiota profiles and health status to maximize efficacy in elderly populations.

## Challenges in analyzing and understanding the gut microbiota composition

Despite significant advancements in gut microbiota research, numerous challenges persist in accurately analyzing and comprehending its composition (Table 3). The characterization of the diverse gut microbiota—consisting of thousands of microbial species—represents a formidable challenge [106]. In addition, each microbial species within the gut microbiota possesses unique metabolic functions and interactions, which can even vary between strains from the same species, adding to the complexity of understanding its composition. This diversity is further influenced by factors such as genetics, diet, lifestyle, and environmental exposures, resulting in substantial inter‐individual variability [1]. Moreover, it is important to note that most of our knowledge regarding the gut microbiota comes from large population studies performed in Western countries [50, 240]. Given the influence that geographical location and socioeconomic factors have on the gut microbiota composition, it is imperative to include more diverse and well‐characterized populations, particularly from developing countries, in future studies [240]. Consequently, establishing a standardized healthy microbiota composition proves challenging [12].

**Table 3:** Key challenges in gut microbiota research.

Challenge	Description
Variability in microbiota composition	Significant differences between individuals and populations, making generalizations difficult
Study design limitations	Short study durations, small sample sizes, and lack of standardization in methodology
Mechanistic gaps	Incomplete understanding of cause‐effect relationships and microbial functions
Inter‐individual differences	Responses to interventions vary greatly, complicating personalized approaches
Ethical considerations	Ethical concerns in FMT, probiotic use, and microbiota‐related therapies

Current techniques for studying the gut microbiota—such as DNA sequencing and metagenomics—have limitations in accurately identifying and quantifying microbial species [241, 242]. Variations in sample collection, processing, and analysis methodologies can introduce biases and affect the reliability of results [241]. Moreover, assigning taxonomic identities to microbial sequences relies on reference databases that may be incomplete or outdated, leading to misclassification and inaccurate characterization of microbial communities [243, 244].

To gain a more accurate picture of the gut microbiota and the role it plays in host health, the metabolic function of individual bacterial strains in the gut microbiota needs to be deciphered. However, understanding the functional capabilities of the gut microbiota requires advanced techniques such as metatranscriptomics and metabolomics [245]. Linking microbial gene expression and metabolite production to specific taxa remains challenging due to the complex interplay of microbial functions within the ecosystem. In addition, only limited data exist on the composition and the function of the fungal communities and viruses within the gut microbiota [1, 14, 15]. Furthermore, the dynamic nature of the gut microbiota composition—influenced by factors such as diet, medication, and host physiology—necessitates longitudinal studies with frequent sampling to accurately capture temporal dynamics [10]. However, conducting such studies is resource‐intensive and logistically challenging.

Another significant challenge in understanding the gut microbiota is the lack of knowledge regarding its development and differentiation along the GI tract, particularly the small intestine. Obtaining samples of the luminal content as well as biopsies from different parts of the intestine is challenging, as it relies on endoscopic procedures [246]. As a result, most studies investigating the gut microbiota rely on fecal samples, which provide limited information about the microbial communities in specific regions of the gut, including the mucus layer. Consequently, there is a gap in our understanding of how the gut microbiota varies along the GI tract and how microbial populations develop and interact in different regions [246].

Although the broad strokes of gut microbiota development are clear, as well as its implications for host health, several challenges persist. For example, further research is needed to decipher the complex interactions between the gut microbiota and the host immune system, epithelial barrier, and other physiological processes. Elucidating these interactions requires interdisciplinary approaches that integrate microbiology, biochemistry, immunology, physiology, and other fields. Additionally, standardizing protocols for gut microbiota analysis and ensuring reproducibility across studies are crucial for advancing the field [241, 242]. Thus, variations in sample collection, processing, and analysis protocols can hinder comparability between studies and pose challenges in interpreting findings.

Addressing these challenges is necessary to gain a more comprehensive understanding of the gut microbiota and will require collaborative efforts across disciplines, technological innovations, and advancements in sampling techniques. By overcoming these obstacles, future research can enhance our understanding of the gut microbiota's role in health and disease and lay the foundation for new targeted interventions. Consequently, unraveling the complexities of the gut microbiota holds the potential to provide new insights into personalized medicine and preventive strategies.

## Conclusions and future projections

The dynamic interplay between the gut microbiota and human health across the lifespan underscores its pivotal role in disease prevention and health promotion. Emerging evidence suggests that interventions, such as probiotics, prebiotics, dietary modifications, and FMT, hold promise in modulating gut microbiota composition to enhance health outcomes. However, the heterogeneity in individual responses and the complexity of gut microbiota dynamics necessitate personalized and precision‐based approaches. For example, more extensive research is needed to further elucidate host‐health benefits of therapies able to modify the composition of the gut microbiota. In addition, understanding the intricate relationship between the gut microbiota and the central nervous system will open for exciting new possibilities for interventions in neurological disorders and mood disturbances.

Longitudinal studies are still few and will be crucial to unravel the aging trajectories of the gut microbiota and its impact on health across the lifespan. By tracking diverse age groups over time, we can uncover insights that may lead to interventions such as microbial‐based therapeutics and dietary approaches aimed at mitigating age‐related diseases. In addition, recognizing the influence of environmental and social factors on gut microbiota composition is important in future studies as initiatives targeting sanitation, access to nutritious foods, and social support systems synergistically can promote gut microbiota resilience and community well‐being.

In conclusion, advancing our understanding of gut microbiota development, stability, and interactions with the host across the lifespan holds the potential to revolutionize preventive and therapeutic approaches for a multitude of diseases. By leveraging cutting‐edge technologies and fostering interdisciplinary collaboration, the future of microbiome research promises to pave the way for personalized medicine, ultimately improving health outcomes and enhancing quality of life across generations.

## Conflict of interest statement

Maria Gloria Dominguez‐Bello holds patent number US10357521B2. Frida Fåk Hållenius is a member of the Scientific Advisory Board of Oriflame AG, holds patent number 10137157, and stocks in ProPrev AB and Neurobiome AB. Katri Korpela has received honoraria as a speaker from Nestlé. Martin Frederik Laursen has received travel support and served as a speaker for Nutricia and Nestlé. John Penders has received grant support from the Dutch Research Council, BMBF Research Initiative for the Conservation of Biodiversity, and the Digestive Foundation, and served as an advisory board member for the INITIALISE project EU. Henrik Munch Roager has received an honorarium for an educational article on the gut microbiota from the Biocodex Microbiota Foundation. Tommi Vatanen has received speaker honoraria from the Nestlé Nutrition Institute. Maria C. Jenmalm has received honoraria for lectures and travel support from BioGaia AB, Danone Nutricia, and Abigo Medical. None of these entities had any influence on the contents of the study. Ida Schoultz, Marcus J. Claesson, Peter Konturek, and Lena Öhman: none.
